# Aging and Chronic Sun Exposure Cause Distinct Epigenetic Changes in Human Skin

**DOI:** 10.1371/journal.pgen.1000971

**Published:** 2010-05-27

**Authors:** Elke Grönniger, Barbara Weber, Oliver Heil, Nils Peters, Franz Stäb, Horst Wenck, Bernhard Korn, Marc Winnefeld, Frank Lyko

**Affiliations:** 1 Research and Development, Beiersdorf AG, Hamburg, Germany; 2 Division of Epigenetics, DKFZ-ZMBH Alliance, German Cancer Research Center, Heidelberg, Germany; 3 Genomics and Proteomics Core Facility, German Cancer Research Center, Heidelberg, Germany; The Babraham Institute, United Kingdom

## Abstract

Epigenetic changes are widely considered to play an important role in aging, but experimental evidence to support this hypothesis has been scarce. We have used array-based analysis to determine genome-scale DNA methylation patterns from human skin samples and to investigate the effects of aging, chronic sun exposure, and tissue variation. Our results reveal a high degree of tissue specificity in the methylation patterns and also showed very little interindividual variation within tissues. Data stratification by age revealed that DNA from older individuals was characterized by a specific hypermethylation pattern affecting less than 1% of the markers analyzed. Interestingly, stratification by sun exposure produced a fundamentally different pattern with a significant trend towards hypomethylation. Our results thus identify defined age-related DNA methylation changes and suggest that these alterations might contribute to the phenotypic changes associated with skin aging.

## Author Summary

Although a role of epigenetic mechanisms in aging and in the adaptation to environmental exposures has been widely assumed, research in this area has been hampered by major methodological challenges. We have now used a novel platform for genome-scale methylation analysis to determine the methylation patterns of human skin samples. Skin represents a particularly suitable model for this study because of its well-known phenotype changes associated with aging and sun exposure, and because skin samples are characterized by a very high degree of cellular homogeneity. By examining 50 samples, and analyzing 50 million data points, we show that aging and sun exposure are associated with comparably small, but significant changes in the DNA methylation patterns of human epidermis and dermis samples. Interestingly, aging was not associated with a general variation in DNA methylation patterns, but rather with a directed DNA hypermethylation shift. Importantly, our results also suggest that epigenetic mechanisms may be functionally important for the phenotypic changes associated with aging and chronic sun exposure.

## Introduction

Aging is defined by characteristic phenotypic changes, but there appear to be few corresponding changes in the genotype. As such, aging represents a fundamental epigenetic phenomenon [1]. Epigenetic mechanisms regulate the interpretation of genetic information and thus have the ability to produce different phenotypes from a single genotype [2]. The corresponding regulatory mechanisms are based on two independent modification systems, the covalent modification of histones and the methylation of cytosine residues in DNA [3]. Over the past few years, numerous histone modifications have been described and it has been suggested that complex histone modification patterns encode detailed information about the regulation of associated genes and promoters [4]. Similarly, the methylation status of a gene promoter can have an important effect on the activity status of the corresponding gene and hypermethylation-associated silencing of tumor suppressor genes has been shown to play a prominent role in human cancers [5].

Epigenetic mechanisms are generally considered to represent a regulatory interface between environmental cues and the genome [6]. More specifically, it has also been suggested that age-associated epigenetic changes might restrict the phenotypic plasticity for a successful adaptation to environmental changes [7]. Due to methodological limitations, however, experimental evidence supporting these notions has remained scarce. Based on the analysis of DNA methylation patterns in peripheral blood samples from monozygotic twins it has been suggested that widespread epigenetic variations arise during the lifetime of human individuals [8]. A later study also described profound methylation changes when unfractionated peripheral blood samples were compared that had been sampled from the same individual 11–16 years apart [9]. However, it was also noted that variations in the cellular composition of tissues are prevalent in older individuals and that the observed epigenetic variations might be a consequence of the tissue heterogeneity [10]. In agreement with this notion, an independent study concluded that tissue-specific interindividual DNA methylation differences are very small and that major variations could only be observed when different tissues were compared [11]. As such, the magnitude of age-related epigenetic changes has remained unresolved.

Human skin represents an organ with several advantages for studying age-associated and environmentally-induced epigenetic changes. The skin is directly exposed to many environmental factors and shows an invariant age-dependent phenotype characterized by changes in the vascular network, reduction in the number of melanocytes and Langerhans cells, decreased thickness of the epidermis, lower levels of specific collagen types and other factors [12], [13]. Skin samples can be obtained from healthy individuals, either as suction blisters or via punch biopsies. Importantly, these samples contain defined tissue layers with a very high degree of cellular homogeneity, with epidermal tissues consisting mainly of keratinocytes and dermal tissues consisting mainly of fibroblasts. We have now used a recently developed array technology [14] for the analysis of complex DNA methylation patterns in skin samples obtained from healthy human volunteers. Our results identify a comparably small, but consistent and statistically significant trend towards DNA hypermethylation in aged samples and consistent hypomethylation of a small set of markers in sun-exposed samples.

## Results

**Figure 1:**
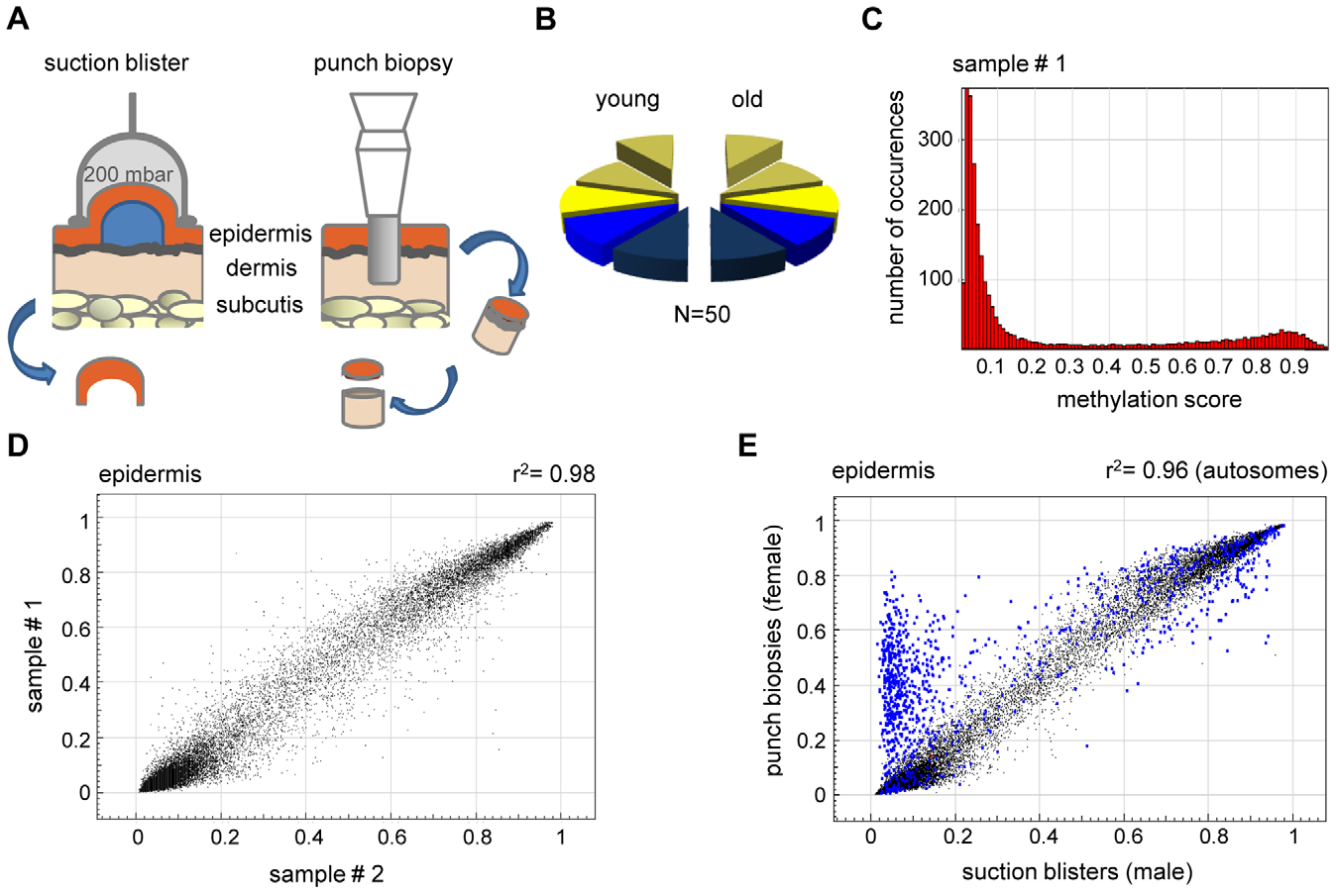
High interindividual similarity between DNA methylation patterns from human epidermis. (A) Procedures for skin sample preparation. Suction blisters were induced on the forearms of healthy volunteers and suction blister roofs were taken as sources for epidermal DNA (left panel). Punch biopsies were obtained from the outer forearm (sun-exposed) and inner arm (sun-protected) and separated into epidermal and dermal layers by dispase II treatment. (B) Schematic outline of the sample groups analyzed in this study. Epidermis (yellow) and dermis (blue) samples were obtained from sun-exposed (bright colors) and sun-protected (shaded colors) skin areas, either as suction blisters (elevated) or as punch biopsies. Each segment represents 5 samples. (C) Representative methylation profile of a human epidermis sample. Most markers were found to be unmethylated (beta<0.2), and a smaller group of markers was found to be highly methylated (beta>0.8). (D) DNA methylation profiles were compared between suction blister samples from two independent young donors. The results show a very high similarity with a correlation coefficient of r^2^ = 0.98. (E) Epidermal DNA methylation profiles were compared between two independent studies. Suction blister samples were obtained from male volunteers, punch biopsy samples were obtained from female individuals. Markers with major variations were almost completely localized to the X-chromosome (marked in blue), and can thus be attributed to the known hypermethylation of X-chromosomal loci in females.

The recent development of robust arrays for comprehensive DNA methylation analysis provided a sensitive tool for the identification of limited DNA methylation changes in the human genome. We investigated DNA methylation patterns in human skin samples, because the tissue is known to undergo well-defined age-related and sun exposure-related phenotypic changes. Skin samples were obtained as suction blisters (epidermis) or as punch biopsies (epidermis and dermis, see Figure 1A). Punch biopsies were taken both from the outer forearm (sun-exposed area) and the inner arm (sun-protected area) and were separated into epidermal and dermal tissues by dispase II treatment. Samples were obtained from healthy male and female donors and from two distinct age groups to analyze the influence of various intrinsic and extrinsic factors on the genomic DNA methylation patterns (Figure 1B, see Table S1 for details). We used Illumina Infinium arrays to determine the methylation status of 27,578 CpG dinucleotides in the human genome. The analysis of 50 samples generated 50 million data points for further analysis (see Materials and Methods for details).

The robustness of array-predicted methylation patterns was characterized by an initial bisulfite sequencing experiment. Three genes with methylation scores (beta values) of 0 (unmethylated), 0.5 (partially methylated) and 1 (completely methylated) were arbitrarily chosen from a pilot array. The analysis of bisulfite sequencing results revealed a very good correlation between the array data and the bisulfite sequencing results for 2 genes (Figure S1). For the partially methylated gene (DIRAS3, Figure S1), bisulfite sequencing indicated a higher methylation level than the array, which can probably be explained by strand-specific PCR bias in the bisulfite sequencing reaction [15]. Indeed, bisulfite sequencing of a second partially methylated gene (ZIM2, Figure S1) showed a very good correlation between the array data and the bisulfite sequencing results. It should be noted that our complete bisulfite sequencing validation set consisted of 10 PCR amplicons (see below in Results) and that bisulfite sequencing confirmed the array-predicted results in 9 out of 10 cases. This strongly suggests that the array produces reliable methylation results.

In an initial step of our analysis, we analyzed methylation profiles from suction blister samples obtained from 5 healthy male donors aged 26–35 years. The methylation profiles (see Figure 1C for an example) showed that the majority (86%) of CpG island associated markers had a methylation score of 0.2 or less and were therefore considered unmethylated. Consistent with other published reports [16], [17], 5% of the CpG island associated markers showed a methylation score of 0.8 or more and were therefore considered methylated. The fraction of methylated markers was substantially higher (29%) in regions not associated with CpG islands, which is again consistent with the general patterns of human DNA methylation reported in other studies [16], [18].

Comparisons between individual methylation profiles also showed a very high degree of similarity between samples (Figure 1D), with correlation coefficients ranging from 0.97–0.98. This substantial similarity of methylation patterns was important for our overall study design, because it permitted the identification of statistically significant changes in a comparably low number of samples. The methylation pattern observed in the pilot sample set obtained from 5 male volunteers aged 26–35 years was largely confirmed in an independent set of epidermis samples obtained from punch biopsies of 5 healthy female individuals aged 19–24 years. However, we also noted a prominent group of markers that had a substantially higher methylation score in the second sample set (Figure 1E). Notably, the vast majority (547 out of 595, Figure 1E) of these markers were derived from the X-chromosome, which is known to be dosage compensated by DNA methylation in females [16], [17], [19]. The observed methylation differences therefore reflect gender differences in the donor groups (Table S1) and further illustrate the sensitivity of the methylation array.

**Figure 2:**
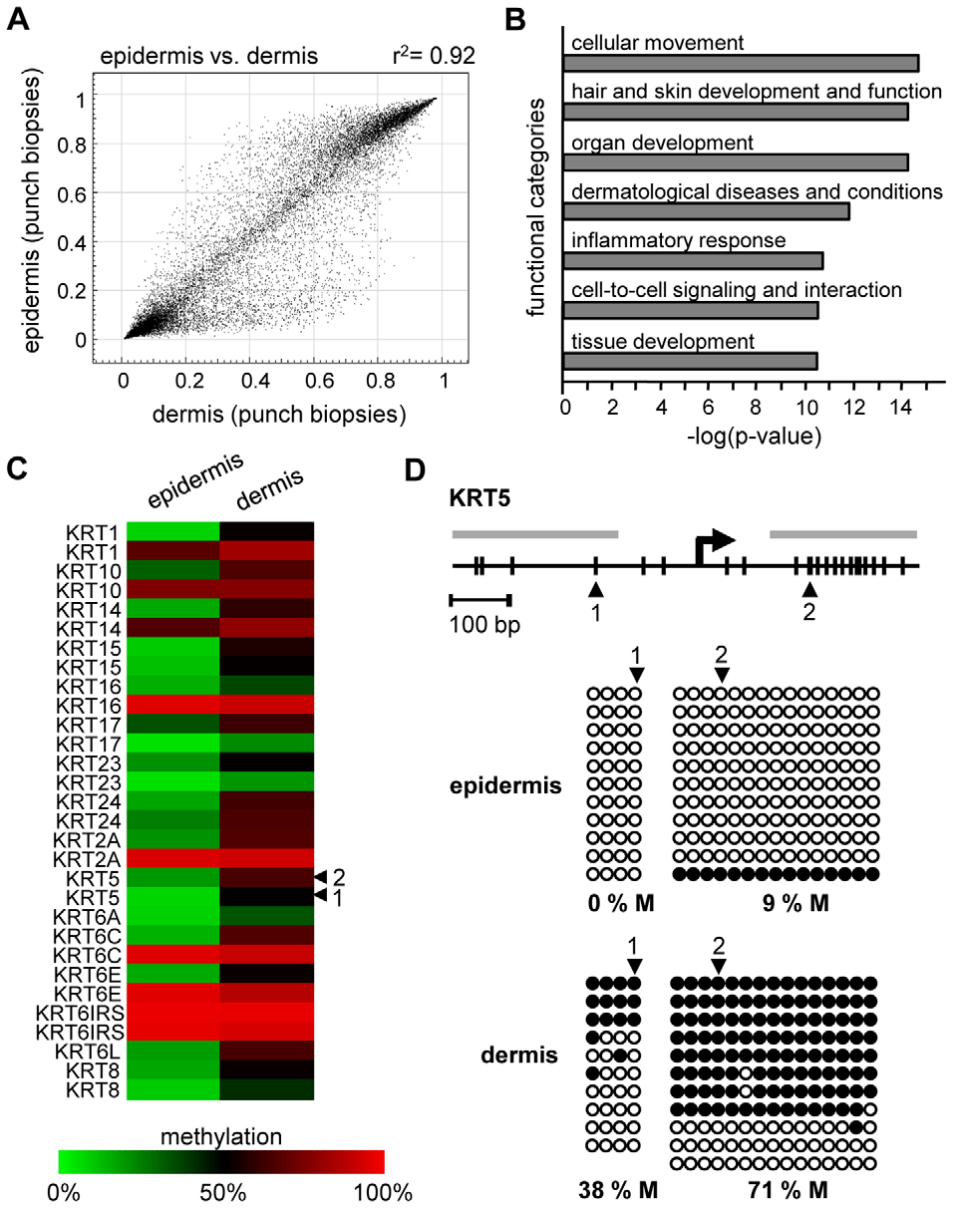
Distinct DNA methylation patterns of human epidermis and dermis. (A) Comparison of average DNA methylation profiles between 20 epidermal and 20 dermal punch biopsy samples. A substantial number of genes show major methylation differences. (B) Ingenuity Pathway Analysis (www.ingenuity.com) of markers with differential (Δ(beta)≥0.2) methylation values in epidermis and dermis. The plot shows the seven most significantly overrepresented functional categories, which are closely associated with (skin) tissue development. Differential methylation of genes associated with “cellular movement” conceivably reflects the prominent differences in the tissue organization between epidermis and dermis. (C) Heatmap of Keratin gene markers with increased (Δ(beta)≥0.2) dermal methylation levels of at least one marker per gene. (D) Validation of differential KRT5 methylation by bisulfite sequencing. Top: Structure of the KRT5 promoter region. CpG dinucleotides are shown as vertical lines, PCR amplicons are shown as grey horizontal bars, the transcription start site (TSS) is indicated by an arrow, numbered arrowheads highlight the CpG sites represented on the Infinium array. Bottom: Bisulfite sequencing results. Each row represents one sequence read, black circles indicate methylated CpG dinucleotides, white circles indicate unmethylated CpG dinucleotides.

We also analyzed the methylation profiles from all dermis samples (n = 20; outer forearm and inner arm) of 10 healthy female individuals aged 18–72 years (Table S1). The results again revealed substantial interindividual similarities, with correlation coefficients between 0.95 and 0.98. However, when the dermal methylation profiles were compared to the matched epidermal methylation profiles of the punch biopsy samples (n = 20), both tissues showed remarkable differences (Figure 2A), with 742 markers being considerably more (Δ(beta)≥0.2) methylated in the epidermis and 1034 markers being more methylated in the dermis. Genes associated with differentially methylated markers were strongly enriched in functional categories associated with the molecular and cellular characteristics of (skin) tissue development (Figure 2B). These findings are consistent with a role of DNA methylation in the regulation of cell type-specific gene expression patterns and illustrate the differences in the cellular composition of epidermis (keratinocytes) and dermis (fibroblasts). In this context, several keratin genes provided a notable example for differential methylation (Figure 2C), which was validated by bisulfite sequencing of the KRT5 gene (Figure 2D). The results showed low levels of KRT5 methylation in the epidermis (0% in the promoter region, 9% in the downstream CpG island) and substantially higher methylation levels in the dermis (38% in the promoter region, 71% in the downstream CpG island). In agreement with a role of DNA methylation in the silencing of cell type-specific genes, KRT5 has been shown to be highly expressed in the epidermis, but not in the dermis [20]–[24].

**Figure 3:**
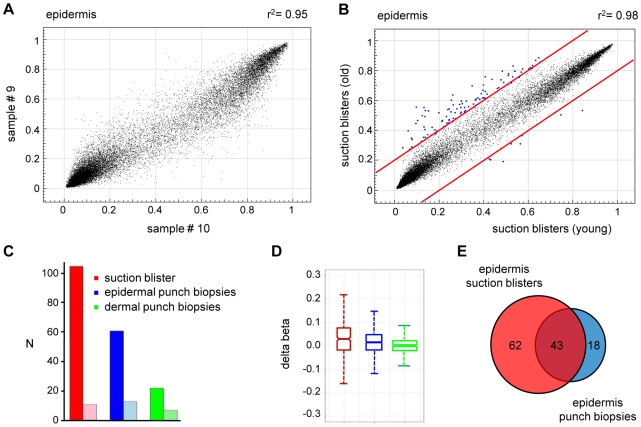
Age-related hypermethylation in human epidermis samples. (A) DNA methylation profiles were compared between epidermal blister samples from two independent old donors. The results show a high similarity with a correlation coefficient of r^2^ = 0.95. (B) Average epidermal DNA methylation profiles of the study population were compared between old and young epidermis samples. The results reveal a distinct trend towards age-associated hypermethylation. (C) Barplot illustrating the results from an independent statistical analysis of the array data. The number of substantially hypermethylated (Δ(beta)≥0.2, P(BH)<0.01) markers is shown in dark colors, the number of substantially hypomethylated (Δ(beta)≤−0.2, P(BH)<0.01) markers is shown in light colors. (D) Boxplot illustrating age-related methylation changes in human epidermis and dermis samples. (E) A comparison of substantially hypermethylated markers from two independent sets of epidermis samples (suction blisters and punch biopsies) reveals a large overlap of 43 commonly hypermethylated markers.

We then extended our analysis to the comparison of methylation patterns of the two distinct age groups. Again, the comparison of suction blister methylation patterns from 5 healthy male individuals aged 65–71 years revealed a very high degree of similarity (Figure 3A), with correlation coefficients between 0.91 and 0.98. The establishment of average methylation values for young and old epidermis samples subsequently allowed the comparison of these data sets. To identify markers with relevant methylation changes, we applied a stringent cutoff at a beta value change of 0.2 or more, based on previously published data [14]. The results indicated that 104 markers (0.37%) showed a beta value increase by 0.2 or more in old epidermis, while only 8 markers (0.03%) showed a beta value decrease by 0.2 or more (Figure 3B). Out of the 104 hypermethylated markers, 90 were associated with CpG islands, which may be related both to the overall low methylation of CpG islands (see above) and to the overrepresentation of CpG island-associated probes on the array [14]. Together, these data suggested that skin aging is associated with predominant hypermethylation in a comparably small set of markers. Age-related hypermethylation was also confirmed by an independent statistical analysis (see Materials and Methods for details) and by inclusion of the methylation profiles from the epidermal and dermal punch biopsy samples (outer forearm and inner arm). The results showed a strong and unambiguous trend towards hypermethylation in epidermis and dermis samples from the old donor group (Figure 3C and 3D). Age-related hypermethylation was stronger in the epidermis than in the dermis (Figure 3C and 3D), which is conceivably due to the more immediate environmental exposure of the epidermis. Out of the 61 markers found to be substantially (Δ(beta)≥0.2, with a Benjamini-Hochberg adjusted P-value P(BH)<0.01) hypermethylated in epidermal punch biopsies from old female individuals, 43 were also found to be hypermethylated in suction blister samples from old male donors, that were obtained with a completely independent sampling protocol (Figure 3E). This result provided further confirmation for a distinct age-associated epigenetic shift in human skin.

**Figure 4:**
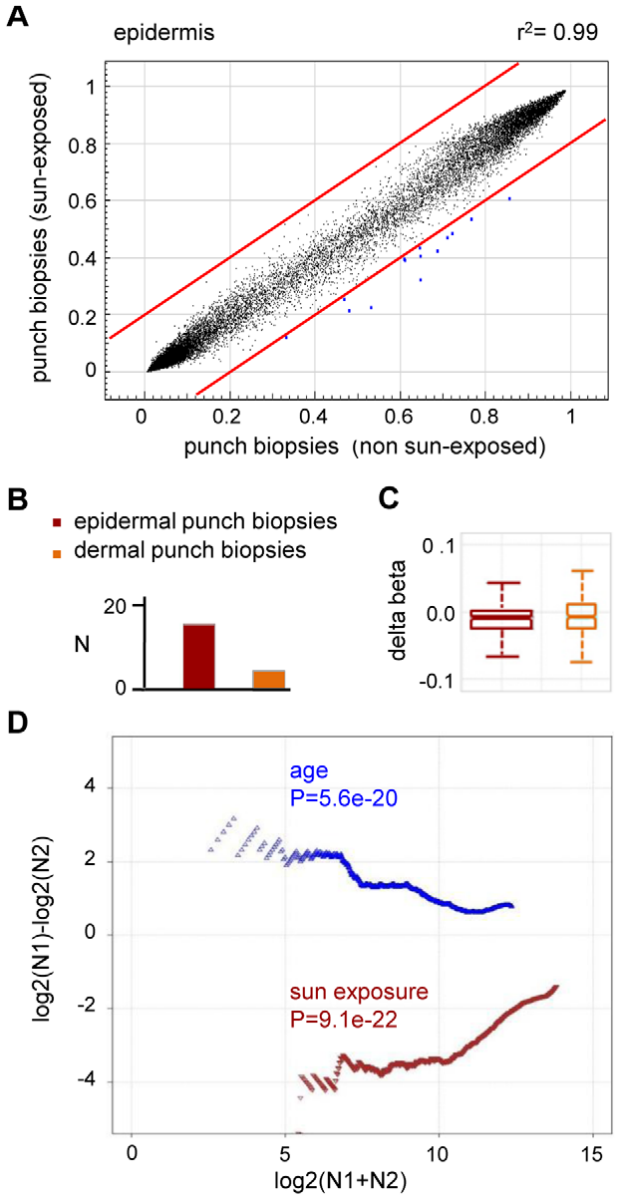
Sun exposure-related hypomethylation. (A) Average epidermal DNA methylation profiles of the study population were compared between 10 sun-exposed and 10 non-exposed epidermis samples. The results reveal a distinct trend towards sun exposure-related hypomethylation. (B) Barplot illustrating the results from an independent statistical analysis of the array data. Bars indicate the number of substantially hypomethylated (Δ(beta)≤−0.2, P(BH)<0.01) markers. (C) Boxplot illustrating sun exposure-related methylation changes in human epidermis and dermis samples. (D) RS (ratio-over-sum) plot illustrating global age-related (blue) and sun exposure-related (brown) methylation shifts in epidermis samples. Points were plotted after Benjamini-Hochberg correction and P-values were obtained after comparisons to Gaussian distributions of randomly generated methylation differences.

To further demonstrate the specificity of the observed methylation shift, we also stratified the punch biopsy sample sets according to chronic sun exposure (outer forearm vs. inner arm). Remarkably, this comparison revealed a fundamentally different methylation change: out of the 27,578 markers analyzed, none showed a beta value increase by 0.2 or more in sun-exposed epidermis, while 14 (0.05%) showed a beta value decrease by 0.2 or more (Figure 4A). Statistical analysis confirmed the trend towards hypomethylation and also indicated that sun exposure-related hypomethylation was less pronounced than age-related hypermethylation (Figure 4B and 4C). Again, the effects appeared stronger in the epidermis than in the dermis (Figure 4B and 4C), which may be related to the more direct sun exposure of the epidermis. Importantly, global age-related and sun exposure-related DNA methylation shifts were highly significant when compared to Gaussian distributions of randomly generated methylation differences and after a global Benjamini-Hochberg adjustment of the P-values (Figure 4D). This finding further confirms our notion that aging and sun exposure cause distinct global DNA methylation changes in human skin.

**Figure 5:**
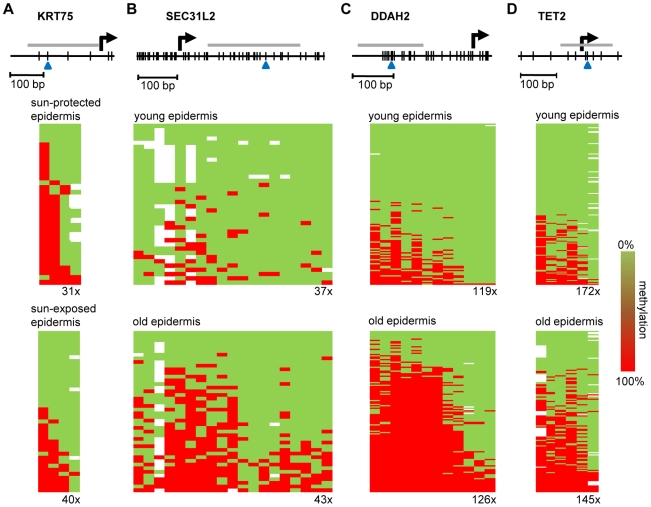
Validation of methylation changes by deep bisulfite sequencing. Schematic outline of the promoter regions of (A) KRT75 (B) SEC31L2, (C) DDAH2, and (D) TET2. Vertical lines represent individual CpG dinucleotides, blue arrowheads indicate CpG markers represented on the array. PCR amplification was performed on equimolar sample pools. PCR amplicons for sequencing are shown as grey horizontal bars, sequencing results are shown as heatmaps. Each row represents one sequence read, individual red boxes represent methylated CpG dinucleotides, green boxes represent unmethylated CpG dinucleotides, sequencing gaps are shown in white. Sequencing coverage ranged from 31x to 172x, as indicated. The deamination efficiency was >99% for all samples analyzed.

Finally, to confirm the observed methylation changes on the level of individual genes we analyzed the gene-specific methylation patterns by bisulfite sequencing. Because previous reports have suggested that UV-induced mutations preferentially occur at methylated CpG dinucleotides [25], a sequencing approach also allowed us to control for the possibility that the methylation changes identified by the array might actually represent genetic polymorphisms or mutations. In a first experiment, we therefore focused on the KRT75 promoter region, which was found among the 16 markers that were substantially (Δ(beta)≤−0.2, P(BH)<0.01) hypomethylated in the sun-exposed epidermis samples. KRT75 encodes a Keratin protein that is normally not expressed in the epidermis, but can affect the intermediate filament architecture of epithelial cells [26], [27]. Results from deep sequencing of pooled samples (epidermis DNA from a sun-protected area and a sun-exposed area from 10 healthy female donors) did not reveal any genetic polymorphisms or mutations in the KRT75 5′ upstream region that could have contributed to the array result (Figure S2). Nevertheless, methylation analysis by deep bisulfite sequencing of the same region confirmed the demethylation of the CpG dinucleotide represented on the array (61% methylation in sun-protected samples, 33% in sun-exposed samples, Figure 5A). Similarly, the data also revealed demethylation for the CpG dinucleotide located immediately distal (81% methylation in sun-protected samples, 48% methylation in sun-exposed samples, Figure 5A). Together, these results validate the demethylation of sun-exposed epidermis samples at the KRT75 promoter region.

Lastly, we also sought to validate the age-associated hypermethylation shift by deep bisulfite sequencing. A probe from the SEC31L2 gene was identified among the 43 markers substantially (Δ(beta)≥0.2, P(BH)<0.01) hypermethylated both in epidermal punch biopsies and in suction blister samples from old individuals. SEC31 proteins have been shown to be important for collagen secretion in primary human fibroblasts [28] and hypermethylation-associated silencing of SEC31L2 could thus play an important role in the age-associated skin phenotype. To experimentally validate age-associated hypermethylation, we pooled equal amounts of epidermis DNA samples obtained from 5 young male (26–35 years) and 5 young female donors (19–24 years) and from 5 older male (65–71 years) and 5 older female (67–72 years) individuals, respectively, and used deep bisulfite sequencing to determine the methylation pattern of 19 CpG dinucleotides in the SEC31L2 5′ region (Figure 5B). Sequence analysis showed that SEC31L2 was mostly unmethylated in the young sample pool, but became distinctly methylated in the old sample pool (Figure 5B). Two additional, arbitrarily chosen loci that showed age-related hypermethylation on the array, DDAH2, an epigenetic marker associated with cellular differentiation [29], [30], and TET2, a putative tumor suppressor gene [31], also were substantially hypermethylated in old epidermis samples (Figure 5C and 5D). These results provide direct confirmation for the array-based findings and again illustrate that aging and sun exposure of human skin are characterized by distinct epigenetic variations.

## Discussion

The molecular pathways contributing to human aging are currently being investigated in many experimental contexts. It is widely assumed that epigenetic changes play a fundamental role in establishing gene expression patterns specific for aged cells and tissues [1]. However, the experimental evidence to support this notion has been limited. For example, it was shown that global DNA methylation levels decrease during in vitro fibroblast cultivation [32], suggesting that DNA hypomethylation might be a molecular marker of aging. More recently, evidence has been provided for age-associated hypermethylation of specific loci in various model systems [1]. However, there are only few published reports that have directly analyzed this question on a genome-scale level.

A distinct age-related phenotype and a high level of cellular homogeneity establish human skin as an excellent model system to study age-related epigenetic alterations. Our results show that defined skin tissue layers (epidermis and dermis) are characterized by specific DNA methylation patterns that are highly similar between individual samples, with correlation coefficients that are commonly achieved for biological and technical replicates. Our results further show that skin aging is associated with DNA hypermethylation in less than 1% of the markers analyzed.

The availability of array-based technologies for methylation patterns analysis also allows the identification of defined epigenetic candidate biomarkers for human aging. For example, a recent study has used Illumina GoldenGate methylation arrays to interrogate the methylation status of 1505 CpG dinucleotides representing 803 cancer-associated genes [33]. While this study investigated age-related methylation changes in various primary human tissues, the analysis was restricted to cancer-associated genes and did not account for the cellular heterogeneity of the tissues analyzed. In addition, the methylation changes appeared rather minor and were only analyzed at single cytosine residues. Our data provides evidence for directed methylation shifts that were validated by bisulfite sequencing of PCR fragments containing several CpG dinucleotides beyond the cytosine residue queried by the array. Notably, the methylation changes observed by bisulfite sequencing of SEC31L2, DDAH2 and TET2 show a distinct similarity to epigenetic mutations described in human cancers [7] (see below).

The molecular steps leading to the establishment of altered methylation patterns are presently unclear. Because DNA methyltransferase mRNA levels did not show any significant differences between old and young and between sun-exposed and non-exposed samples (Figure S3), the observed DNA methylation changes do not seem to involve altered expression of DNA methyltransferase genes. An association between DNA methylation and sunlight-mediated mutagenesis has been suggested previously, based on the observation that UV-induced mutations preferentially occurred at methylated CpG dinucleotides [25]. However, when we analyzed the mutational status of KRT75 by deep sequencing, we did not detect any evidence for local genetic mutations. It is possible that the observed epigenetic changes may be influenced by more distant genetic mutations or by mutations in trans-acting epigenetic modifiers. These details will have to be investigated in future studies.

Recently, specific age-related hypermethylation has also been described at a subset of developmentally regulated genes in various human tissues [34], [35]. While the methylation differences observed in these studies were highly significant, they were also comparably small and might thus have escaped detection in our analysis. In addition, age-related hypermethylation has also been observed in the human intestine [36], [37], and has been interpreted as a preneoplastic epigenetic lesion. Age is the most significant risk factor for human cancer [38] and gene-specific hypermethylation represents one of the most consistent markers of tumorigenesis [5]. Our bisulfite sequencing results showed pronounced age-related hypermethylation of DDAH2, which encodes a key enzyme in the nitric oxide pathway. Nitric oxide plays an important role in the regulation of keratinocyte proliferation and in the development of skin cancer [39]. Similarly, our results also demonstrated age-related hypermethylation of TET2, a putative tumor suppressor gene that has recently been shown to be genetically mutated in myeloproliferative disorders [31]. These findings lend further support to the notion that age-related epigenetic changes provide a molecular link between aging and tumorigenesis.

## Materials and Methods

### Ethics statement

The recommendations of the current version of the Declaration of Helsinki and the guideline of the International Conference on Harmonization Good Clinical Practice (ICH GCP) were observed as applicable to a non-drug study. All donors provided written, informed consent. Punch biopsies were obtained through a clinical study approved by the Ethics Committee of the Medical Association of Hamburg (PV 3107). All volunteers provided written, informed consent.

### Tissue samples

Suction blisters were obtained from the volar forearms of 10 healthy male volunteers, as described previously [40]. Suction blister roofs were taken and immediately stored at −20°C. Full-thickness skin samples (diameters: 6 mm or 4 mm) were obtained from 20 female volunteers by Sciderm GmbH (Hamburg, Germany). Biopsies were isolated from the outer forearm (sun-exposed area) and inner arm (sun-protected area), respectively. Immediately after removal, punch biopsies were transferred into DMEM medium (Gibco BRL) and stored on ice for up to 5 h. After dispase II treatment (2 U/ml, Roche) for 2 h at 37°C, epidermis and dermis were separated and stored at −80°C.

### DNA and RNA isolation

Suction blister and punch biopsy samples (epidermis or dermis) were washed in DPBS (Cambrex) and homogenized using a TissueLyser (Retsch). DNA from suction blister samples was isolated using the QIAamp DNA Investigator Kit (Qiagen) according to the manufacturer's instructions. RNA and DNA from punch biopsy samples were processed with the Qiagen AllPrep DNA/RNA/Protein Mini Kit (Qiagen) as recommended by the supplier. The concentrations and purities of isolated DNA and RNA were assessed spectrophotometrically using a NanoDrop ND-1000 (Peqlab).

### Array-based methylation analysis

The HumanMethylation27 BeadChip has been described previously [14]. A single BeadChip utilizes more than 1,000,000 beads per sample and generates 27,578 DNA methylation measurements. The CpGs under investigation are located in more than 13,500 promoters of well-annotated genes. Genomic DNA (500 ng) from each sample was bisulfite converted using the EZ-96 DNA Methylation Kit (Zymo Research Corporation) according to the manufacturer's recommendations. After bisulfite conversion, each sample was whole-genome amplified, enzymatically fragmented, and about 200 ng of DNA was applied to the arrays. During hybridization, the DNA molecules anneal to locus-specific DNA oligomers linked to individual bead types. After extension, the array was fluorescently stained, scanned, and the intensities of the non-methylated and methylated bead types were measured. DNA methylation values, described as beta values, were recorded for each locus in each sample and analyzed by BeadStudio (Illumina).

### Data deposition

Microarray data are available in the ArrayExpress database (www.ebi.ac.uk/arrayexpress) under accession number E-MTAB-202.

### Statistical analysis of array data

Because the raw data from the green and the red channel of the array did not show the same shape of distribution, both channels were quantile normalized [41] separately. Subsequently, all beads of one probeID from all samples of a group were aggregated to calculate the mean values, standard deviations, detection P-values and beta values of the given probeID in the group. The detection P-value is defined as the minimum of the 2 separate P-values of the 2 variables (grn.A/B or red.A/B), where each P-value is the result of testing (Mann-Whitney-U-test) against the negative beads of each channel. Because 2 variables were measured to calculate the beta value we applied the Benjamini-Hochberg correction [42] before choosing the minimum P-value. Less than 0.1% of the probeIDs showed detection P-values >0.05 and the corresponding data were excluded from further analysis.

For differential methylation analysis, the difference of beta values was calculated by using the mean beta values of the two groups in question and the two-sample Wilcoxon test (Mann-Whitney-U-test) was used to calculate P-values. For global statements about overall methylation effects all P-values were adjusted using the Benjamini-Hochberg [42] correction.

To visualize the differential methylation trend between two groups the data points were plotted as an RS-plot (ratio over sum plot), which is a variation of the MA-plot [43]. Each point was calculated as follows: Calculate the difference (Δ(beta)) between the beta values of two groups and sort the data according to the absolute difference in decreasing order. The N-th data point for the graph is then calculated as: 
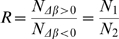
and 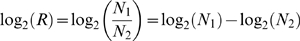 on the y-Axis

and 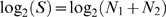 on the x-Axis.

Assuming a normal distribution of Δ(beta) (P(BH)<0.01) values, the t-test was used to test the distributions of the experimental data (sd<0.1) against a random normal distribution (mean = 0.0, sd = 0.1). This provided P-values for the significance of the observed global methylation shifts.

### Bisulfite sequencing

Genes were randomly chosen from array-predicted groups. For bisulfite treatment of DNA, the EpiTect Bisulfite Kit (Qiagen) was used according to the manufacturer's instructions. Up to 300 ng DNA was used. Deaminated DNA was amplified by PCR using the following primers: Krt5_1_for GTTGTTTGGAAAAGTGTAAGAGTAGATTAT, Krt5_1_rev TACCTTACAACACTAATCTCTTAACAACAA, Krt5_2_for AGTGTTTGGTTTTTTTGTTTTATTAGG, Krt5_2_rev CCCCCAAATTATAAAAACTCC. PCR conditions were as follows: 95°C for 3 min followed by 40 cycles at 95°C for 30 sec, annealing temperature for 40 sec and 72°C for 45 sec. At last, the reaction was incubated at 72°C for 3 min. PCR products were gel-extracted using the QIAquick gel extraction Kit (Qiagen) and cloned using the TOPO TA cloning Kit for sequencing (Invitrogen), according to the manufacturer's instructions.

### Deep bisulfite sequencing

Equal amounts of DNA from each skin sample were pooled according to age and sun exposure, respectively. The DNA pools were bisulfite treated by using the EpiTect Bisulfite Kit (Qiagen) according to the manufacturer's instructions. KRT75, DDAH2, TET2 and SEC31L2 fragments were amplified by PCR, using the primers KRT75_for GGTTTGTATTAATATAAGATGTTTGGATAG, KRT75_rev AACCACTAACTAATTCCCTAACACC, DDHA2_ for TAGGGTAGAAGTTAGGAATTAAGAAGG, DDAH2_rev CCAAACCCACCCAAATCTAA, TET2_for GAGAAATTTATTTTAATTTGTGAGA, TET2_rev TAAAAACCTATATTTTTAAAAACCC, SEC31L2_for GTTTGGGGTTTTTGGTAGTAGAGA, SEC31L2_rev CAACAATAAACAAAAAAACCCTCAT. PCR conditions were as follows: 95°C for 3 min followed by 40 cycles at 95°C for 30 sec, annealing temperature for 40 sec and 72°C for 45 sec, followed by a 3 min incubation at 72°C. PCR products were subsequently purified using the QIAquick gel extraction Kit (Qiagen). For sequencing, equimolar amounts of all amplicons were combined in a single tube. Ligation of adaptor sequences and pool-specific tags and Roche 454 sequencing was provided by GATC (Konstanz, Germany) and LGC Genomics (Berlin, Germany). Sequencing data were processed using DNAstar Lasergene 8.0 software.

## Supporting Information

Figure S1 Validation of array-predicted methylation levels of ANP32E, DIRAS3, ZIM2, and MGMT by bisulfite sequencing. DNA was treated with sodium bisulfite by using the Qiagen EpiTect bisulfite kit. Converted DNA was amplified by PCR using the following primers: ANP32E_for TATTTTTTTAGGGGGTGGGTTTTTT, ANP32E_rev CTTAATCAAACAACAACAAAAAAAA; DIRAS3_for TATTTTAATAGGTGAGAAAAAGTTTATAGT, DIRAS3_rev ACCAAACAACCTAAAAAACAAATAC; ZIM2_for GGGGTAAGGTTGAAGTGGTTGTAGG, ZIM2_rev CCAAACTAAAATTCATAAAATTACC; MGMT_for GTTTTTTGTGATTGGTTTATTTTATG, MGMT_rev ACCAAAAACACACTCTAACAATCTC. PCR conditions were as follows: 95°C for 3 min followed by 40 cycles at 95°C for 30 sec, annealing temperature for 40 sec and 72°C for 45 sec. At last, the reaction was incubated at 72°C for 3 min. PCR products were gel extracted using the QIAquick gel extraction Kit (Qiagen) and cloned using the TOPO TA cloning kit for sequencing (Invitrogen).
(3.30 MB TIF)

Figure S2 Genetic analysis of the KRT75 promoter region. The PCR amplicon for sequence analysis is shown as a grey horizontal bar, vertical lines represent individual CpG dinucleotides (also see Figure 5A). DNA was amplified by PCR using the primers KRT75 (genomic)_for AGGAAGCACCCCAAGGAAAC, KRT75 (genomic)_rev ACGTGCAAACTCCTTTCCAG. PCR, sequencing and analysis were performed as described for deep bisulfite sequencing. The heatmaps show the absence of genetic polymorphisms at these CpG dinucleotides and the sequencing coverage is indicated next to the heatmaps.
(11.88 MB TIF)

Figure S3 Levels of DNMT mRNA expression in various epidermis samples. Total RNA was reverse transcribed using the High Capacity cDNA Reverse Transcription kit (Applied Biosystems), according to the manufacturer's instructions. The resulting cDNA was analyzed for mRNA expression of DNMT1, DNMT3a and DNMT3b by Real-Time TaqMan-PCR using the 7900HT Fast-Real-Time PCR System (Applied Biosystems). FAM labelled primers for the qRT-PCR (Applied Biosystems) were Inventoried TaqMan Assays for glyceraldehyde-3-phosphate dehydrogenase (GAPDH; Hs99999905_m1) and for DNMTs (DNMT1: Hs00154749_m1, DNMT3a: Hs01027166_m1, DNMT3b: Hs01003405_m1). PCR conditions were as follows: 95°C for 20 sec followed by 40 cycles at 95°C for 1 sec and 60°C for 20 sec. Real-time PCR data were analyzed using the Sequence Detector (Version 2.3) software supplied with the 7900HT Fast-Real-Time PCR System (Applied Biosystems). Quantification was achieved by calculating the relative changes in gene expression of the target normalized to an endogenous reference (GAPDH) and relative to the average of all delta Ct-values. The results show no significant differences in DNMT mRNA expression levels between old/young and sun-exposed/non-exposed epidermis samples.
(1.10 MB TIF)

Table S1 Tissue samples used in this study.
(0.08 MB DOC)
