# Supplementary figures and images for: Aging and Chronic Sun Exposure Cause Distinct Epigenetic Changes in Human Skin
Source: PLoS Genet. 2010 May 27;6(5):e1000971. doi: 10.1371/journal.pgen.1000971 (PMC2877750; doi:10.1371/journal.pgen.1000971)

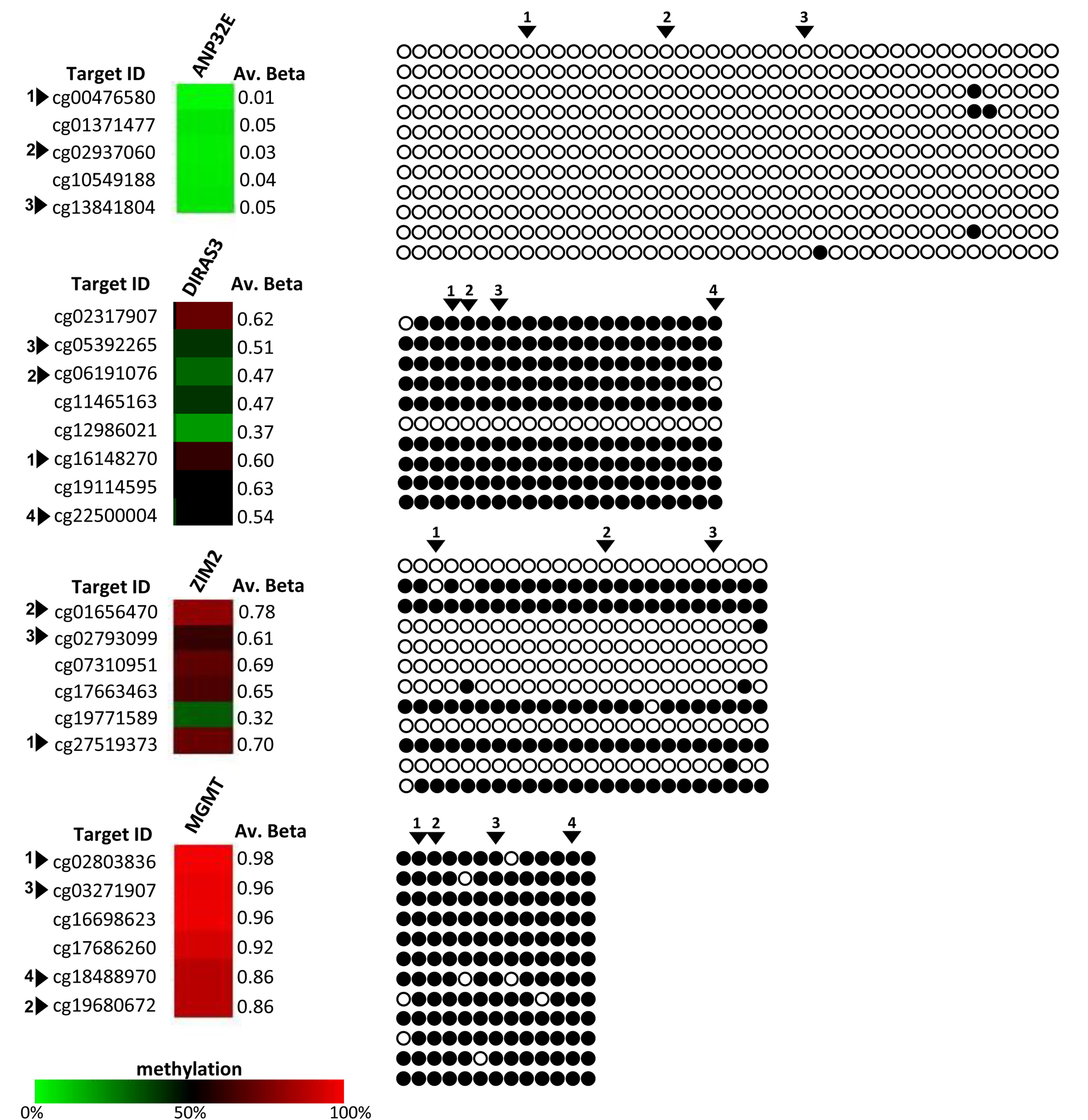

Supplement: Figure S1 — Validation of array-predicted methylation levels of ANP32E, DIRAS3, ZIM2, and MGMT by bisulfite sequencing. DNA was treated with sodium bisulfite by using the Qiagen EpiTect bisulfite kit. Converted DNA was amplified by PCR using the following primers: ANP32E_for TATTTTTTTAGGGGGTGGGTTTTTT, ANP32E_rev CTTAATCAAACAACAACAAAAAAAA; DIRAS3_for TATTTTAATAGGTGAGAAAAAGTTTATAGT, DIRAS3_rev ACCAAACAACCTAAAAAACAAATAC; ZIM2_for GGGGTAAGGTTGAAGTGGTTGTAGG, ZIM2_rev CCAAACTAAAATTCATAAAATTACC; MGMT_for GTTTTTTGTGATTGGTTTATTTTATG, MGMT_rev ACCAAAAACACACTCTAACAATCTC. PCR conditions were as follows: 95°C for 3 min followed by 40 cycles at 95°C for 30 sec, annealing temperature for 40 sec and 72°C for 45 sec. At last, the reaction was incubated at 72°C for 3 min. PCR products were gel extracted using the QIAquick gel extraction Kit (Qiagen) and cloned using the TOPO TA cloning kit for sequencing (Invitrogen). (3.30 MB TIF) [file pgen.1000971.s001.tif]

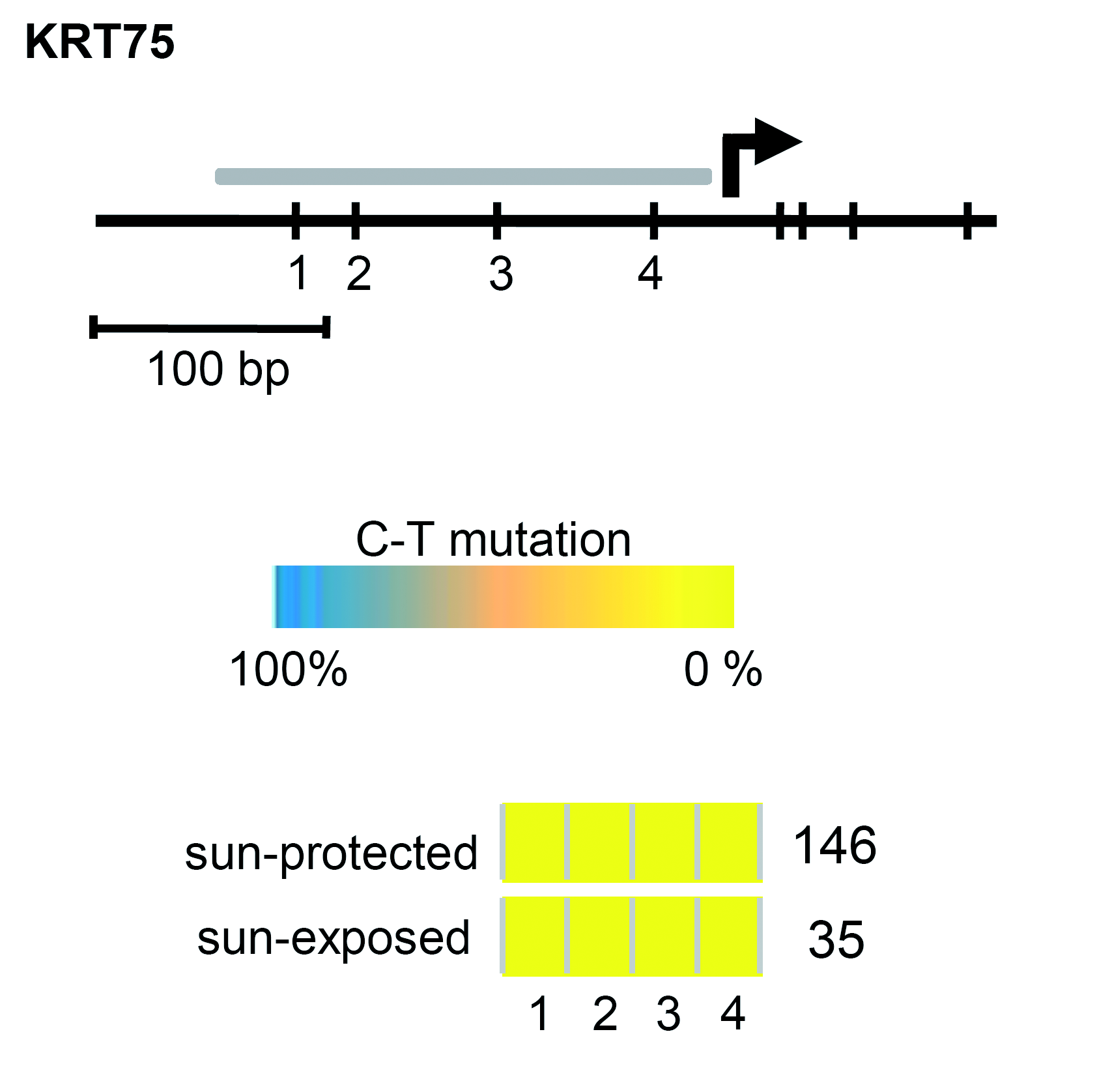

Supplement: Figure S2 — Genetic analysis of the KRT75 promoter region. The PCR amplicon for sequence analysis is shown as a grey horizontal bar, vertical lines represent individual CpG dinucleotides (also see Figure 5A). DNA was amplified by PCR using the primers KRT75 (genomic)_for AGGAAGCACCCCAAGGAAAC, KRT75 (genomic)_rev ACGTGCAAACTCCTTTCCAG. PCR, sequencing and analysis were performed as described for deep bisulfite sequencing. The heatmaps show the absence of genetic polymorphisms at these CpG dinucleotides and the sequencing coverage is indicated next to the heatmaps. (11.88 MB TIF) [file pgen.1000971.s002.tif]

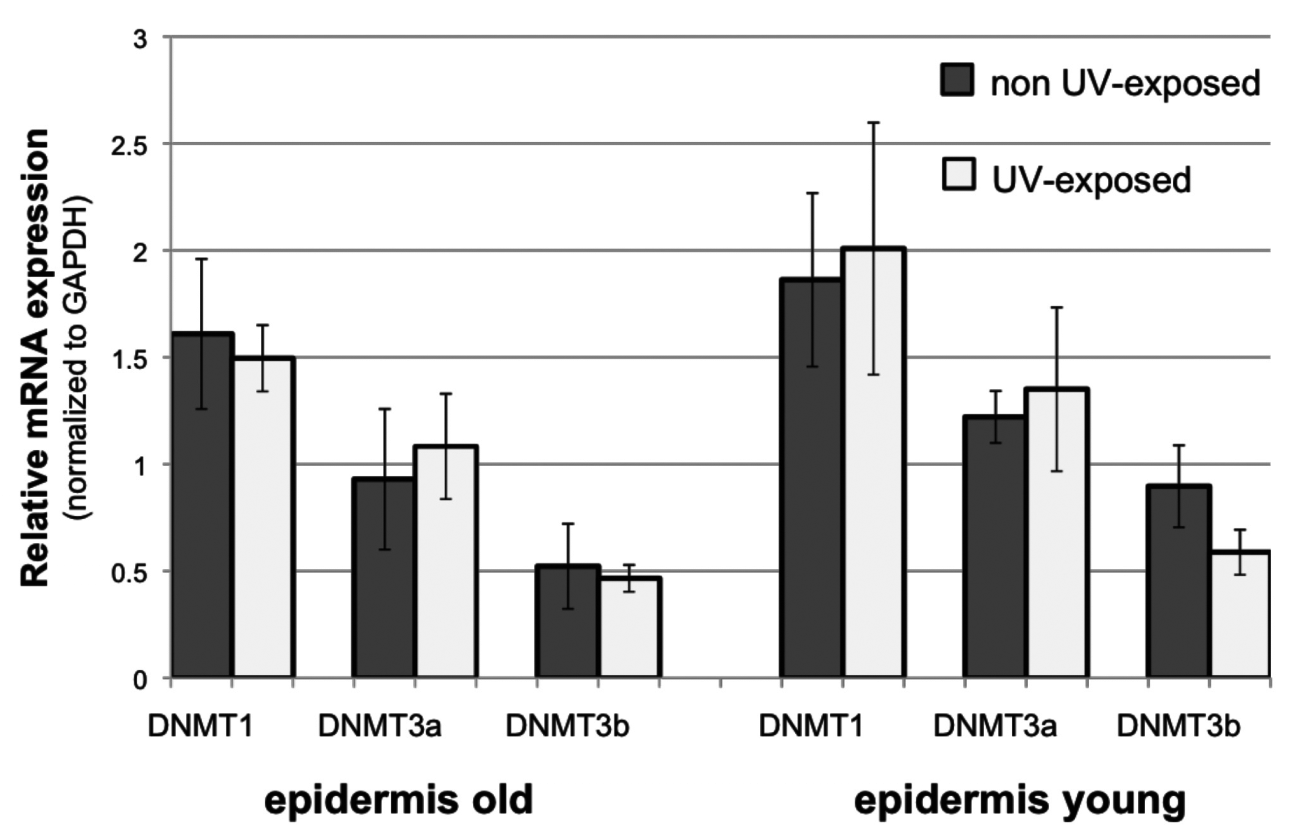

Supplement: Figure S3 — Levels of DNMT mRNA expression in various epidermis samples. Total RNA was reverse transcribed using the High Capacity cDNA Reverse Transcription kit (Applied Biosystems), according to the manufacturer's instructions. The resulting cDNA was analyzed for mRNA expression of DNMT1, DNMT3a and DNMT3b by Real-Time TaqMan-PCR using the 7900HT Fast-Real-Time PCR System (Applied Biosystems). FAM labelled primers for the qRT-PCR (Applied Biosystems) were Inventoried TaqMan Assays for glyceraldehyde-3-phosphate dehydrogenase (GAPDH; Hs99999905_m1) and for DNMTs (DNMT1: Hs00154749_m1, DNMT3a: Hs01027166_m1, DNMT3b: Hs01003405_m1). PCR conditions were as follows: 95°C for 20 sec followed by 40 cycles at 95°C for 1 sec and 60°C for 20 sec. Real-time PCR data were analyzed using the Sequence Detector (Version 2.3) software supplied with the 7900HT Fast-Real-Time PCR System (Applied Biosystems). Quantification was achieved by calculating the relative changes in gene expression of the target normalized to an endogenous reference (GAPDH) and relative to the average of all delta Ct-values. The results show no significant differences in DNMT mRNA expression levels between old/young and sun-exposed/non-exposed epidermis samples. (1.10 MB TIF) [file pgen.1000971.s003.tif]
