# Supplementary material for: Aging and Chronic Sun Exposure Cause Distinct Epigenetic Changes in Human Skin
Source: PLoS Genet. 2010 May 27;6(5):e1000971. doi: 10.1371/journal.pgen.1000971 (PMC2877750; doi:10.1371/journal.pgen.1000971)
Supplement: Table S1 — Tissue samples used in this study. (0.08 MB DOC) [file pgen.1000971.s004.doc]

Table 1. Tissue samples used in this study.

| **sample no.** | **tissue** | **location** | **age** | **gender** |
| --- | --- | --- | --- | --- |
| 1 | epidermis (suction blister) | inner forearm | 26 | male |
| 2 | epidermis (suction blister) | inner forearm | 26 | male |
| 3 | epidermis (suction blister) | inner forearm | 34 | male |
| 4 | epidermis (suction blister) | inner forearm | 35 | male |
| 5 | epidermis (suction blister) | inner forearm | 35 | male |
| 6 | epidermis (suction blister) | inner forearm | 66 | male |
| 7 | epidermis (suction blister) | inner forearm | 65 | male |
| 8 | epidermis (suction blister) | inner forearm | 69 | male |
| 9 | epidermis (suction blister) | inner forearm | 71 | male |
| 10 | epidermis (suction blister) | inner forearm | 68 | male |
| 11 | epidermis (punch biopsy) | outer forearm | 67 | female |
| 12 | epidermis (punch biopsy) | inner arm |  | female |
| 13 | epidermis (punch biopsy) | outer forearm | 72 | female |
| 14 | epidermis (punch biopsy) | inner arm |  | female |
| 15 | epidermis (punch biopsy) | outer forearm | 67 | female |
| 16 | epidermis (punch biopsy) | inner arm |  | female |
| 17 | epidermis (punch biopsy) | outer forearm | 69 | female |
| 18 | epidermis (punch biopsy) | inner arm |  | female |
| 19 | epidermis (punch biopsy) | outer forearm | 68 | female |
| 20 | epidermis (punch biopsy) | inner arm |  | female |
| 21 | epidermis (punch biopsy) | outer forearm | 23 | female |
| 22 | epidermis (punch biopsy) | inner arm |  | female |
| 23 | epidermis (punch biopsy) | outer forearm | 19 | female |
| 24 | epidermis (punch biopsy) | inner arm |  | female |
| 25 | epidermis (punch biopsy) | outer forearm | 22 | female |
| 26 | epidermis (punch biopsy) | inner arm |  | female |
| 27 | epidermis (punch biopsy) | outer forearm | 22 | female |
| 28 | epidermis (punch biopsy) | inner arm |  | female |
| 29 | epidermis (punch biopsy) | outer forearm | 24 | female |
| 30 | epidermis (punch biopsy) | inner arm |  | female |
| 31 | dermis (punch biopsy) | outer forearm | 65 | female |
| 32 | dermis (punch biopsy) | inner arm |  | female |
| 33 | dermis (punch biopsy) | outer forearm | 67 | female |
| 34 | dermis (punch biopsy) | inner arm |  | female |
| 35 | dermis (punch biopsy) | outer forearm | 68 | female |
| 36 | dermis (punch biopsy) | inner arm |  | female |
| 37 | dermis (punch biopsy) | outer forearm | 72 | female |
| 38 | dermis (punch biopsy) | inner arm |  | female |
| 39 | dermis (punch biopsy) | outer forearm | 64 | female |
| 40 | dermis (punch biopsy) | inner arm |  | female |
| 41 | dermis (punch biopsy) | outer forearm | 19 | female |
| 42 | dermis (punch biopsy) | inner arm |  | female |
| 43 | dermis (punch biopsy) | outer forearm | 22 | female |
| 44 | dermis (punch biopsy) | inner arm |  | female |
| 45 | dermis (punch biopsy) | outer forearm | 18 | female |
| 46 | dermis (punch biopsy) | inner arm |  | female |
| 47 | dermis (punch biopsy) | outer forearm | 24 | female |
| 48 | dermis (punch biopsy) | inner arm |  | female |
| 49 | dermis (punch biopsy) | outer forearm | 22 | female |
| 50 | dermis (punch biopsy) | inner arm |  | female |
